# Thermal and Structural Analysis of a High-Entropy Cr_16_Mn_16_Fe_16_Co_16_Ni_16_P_20_ Alloy—Influence of Cooling Rates on Phase Transformations

**DOI:** 10.3390/ma17235772

**Published:** 2024-11-25

**Authors:** Krzysztof Ziewiec, Artur Błachowski, Krystian Prusik, Marcin Jasiński, Aneta Ziewiec, Mirosława Wojciechowska

**Affiliations:** 1 Institute of Technology, University of the National Education Commission (UKEN), ul. Podchorążych 2, 30-084 Krakow, Poland; 2 AGH University of Krakow, Faculty of Geology, Geophysics and Environmental Protection, al. A. Mickiewicza 30, 30-059 Krakow, Poland; 3 Faculty of Science and Technology, Institute of Materials Engineering, University of Silesia (UŚ), 75 Pułku Piechoty Street 1A, 41-500 Chorzów, Poland; 4 AGH University of Krakow, Faculty of Metals Engineering and Industrial Computer Science, al. A. Mickiewicza 30, 30-059 Krakow, Poland

**Keywords:** high-entropy alloy, cooling rate, phase transformations, amorphization, phosphorus, microstructure, Mössbauer spectroscopy, X-ray diffraction, thermal analysis

## Abstract

This study investigates the influence of cooling rates on the microstructure and phase transformations of the high-entropy alloy Cr_16_Mn_16_Fe_16_Co_16_Ni_16_P_20_. The alloy was synthesized via arc melting and subjected to three cooling conditions: slow cooling (52 K/s), accelerated cooling after a short electric arc pulse (3018 K/s), and rapid quenching (10⁵–10⁶ K/s) using the melt-spinning method. The microstructures were characterized using X-ray diffraction (XRD), scanning electron microscopy (SEM), transmission electron microscopy (TEM), and Mössbauer spectroscopy. The thermal properties and phase transformations were analyzed using differential scanning calorimetry (DSC) and thermography. Slow cooling produced a complex crystalline microstructure, while accelerated cooling resulted in fewer phases. Rapid cooling yielded an amorphous structure, demonstrating that phosphorus and high mixing entropy enhance glass-forming ability. Phase transformations exhibited significant undercooling under accelerated cooling, with FCC phase crystallization shifting from 1706 K (slow cooling) to 1341 K, and eutectic crystallization from 1206 K to 960 K. These findings provide a foundation for optimizing cooling processes in high-entropy alloys for advanced structural and functional applications.

## 1. Introduction

High-entropy alloys (HEAs) are an innovative class of materials that have gained substantial interest in recent decades due to their unique structural and functional properties, which surpass those of conventional alloys in several applications [1,2]. Unlike traditional alloys, which are often based on one or two main elements, HEAs typically comprise five or more principal elements in near-equimolar ratios. This high configurational entropy stabilizes simple solid-solution phases, thereby enhancing the alloy’s thermal stability and resistance to wear, corrosion, and mechanical stress [3,4,5]. While many studies have focused on traditional high-entropy alloys, there is a limited understanding of how phosphorus addition combined with varied cooling rates influences phase transformations and microstructural development in HEAs. This study aims to address this gap by systematically investigating the Cr_16_Mn_16_Fe_16_Co_16_Ni_16_P_20_ alloy under different cooling conditions. The discovery of the equimolar CrMnFeCoNi alloy, also known as the Cantor alloy, has inspired extensive research on HEAs due to its exceptional combination of strength and ductility across a wide temperature range [6,7,8,9,10]. The potential of HEAs extends beyond their crystallized forms. Recent studies have explored the formation of metallic glasses (MGs) within the HEA framework, revealing exceptional mechanical properties, such as ultra-high strength and remarkable wear and corrosion resistance [11,12,13]. This has led to the development of high-entropy metallic glasses (HEMGs), where the high mixing entropy combined with an amorphous structure yields materials with enhanced glass-forming ability (GFA) and mechanical stability [11,13,14]. In particular, the addition of phosphorus has been shown to significantly support amorphization processes, thereby promoting the formation of stable metallic glass structures [15,16].

A notable factor influencing HEA properties is the cooling rate during solidification. Recent investigations, such as those on the Al0.5CoCrFeNi HEA, demonstrate that varying the cooling rate alters the phase composition and mechanical response, shifting phase equilibria and affecting properties such as hardness, tensile strength, and elongation [17]. This insight is essential for optimizing HEA production and tailoring their properties to specific industrial applications. Despite the benefits of phosphorus addition and high cooling rates, such as improved amorphization, these factors may introduce limitations. High cooling rates can induce internal stresses or microstructural inhomogeneities, potentially compromising ductility. Similarly, while phosphorus enhances glass-forming ability, it may reduce toughness or thermal stability in some cases. Understanding these trade-offs is critical for optimizing HEAs for targeted applications. Studies on mechanical alloying and consolidation processes further highlight the role of processing conditions in refining HEA microstructures, with techniques like spark plasma sintering (SPS) offering superior homogeneity and nanoscale crystallinity.

The unique properties of phosphorus-containing amorphous alloys inspired the research presented in this study. Phosphorus contributes to the formation of stable protective layers, significantly enhancing corrosion resistance in challenging environments [18]. Additionally, phosphorus improves hardness and wear resistance under mechanical load, making alloys such as Ni-P exceptionally durable [19]. Its inclusion in Ni-Fe-Cu-P-Si-B alloys also enables the development of extremely soft magnetic materials, broadening the potential applications of these alloys [20]. Furthermore, recent findings on NiNbP bulk metallic glasses highlight how phosphorus additions enhance glass-forming ability and increase hardness, albeit at the cost of some ductility [21]. Inspired by these results, this study explores the microstructure, mechanical behavior, and phase stability of phosphorus-containing alloys to better understand and utilize their unique properties. The combination of high configurational entropy and phosphorus enrichment in these alloys suggests their potential for applications in demanding environments that require enhanced wear resistance, corrosion resistance, and thermal stability. These properties position them as promising candidates for use in the aerospace, automotive, and tooling industries, as well as in advanced coating technologies and high-performance structural components. What sets this study apart is the focus on how varying cooling rates, combined with phosphorus addition, influence not only amorphization but also the resulting phase transformations and their potential implications for mechanical and magnetic properties. This study provides a detailed analysis that has not been previously reported for the Cr_16_Mn_16_Fe_16_Co_16_Ni_16_P_20_ system.

Despite extensive studies on HEAs, research focusing on phosphorus-enriched HEAs under varied cooling conditions remains limited. This study bridges this gap, providing insights into the mechanisms of amorphization and phase transformation influenced by rapid cooling rates. This study focuses on the high-entropy alloy Cr_16_Mn_16_Fe_16_Co_16_Ni_16_P_20_, selected for its anticipated amorphization potential resulting from high configurational entropy and phosphorus addition. The cooling rates were systematically varied, with slow cooling averaging approximately 52 K/s, accelerated cooling at 3018 K/s, and melt-spinning achieving rapid cooling rates in the range of 10⁵–10⁶ K/s. We investigated the alloy’s phase transformations and microstructural evolution under different cooling conditions, including slow cooling on a copper plate, accelerated cooling after remelting, and rapid quenching using melt-spinning. By characterizing the resulting structures using XRD, SEM, TEM, and Mössbauer spectroscopy, we aim to advance the understanding of processing-condition effects on phase stability and amorphization in phosphorus-enriched HEAs, providing insights for the development of HEAs with tailored properties. Moreover, this study seeks to quantify the extent of undercooling and its influence on phase formation, providing a pathway to optimize both phosphorus composition and cooling rates for balanced mechanical and thermal properties in HEAs. The observed phase transformation temperatures and associated thermal anomalies provide crucial insights into the crystallization mechanisms in phosphorus-enriched high-entropy alloys. These findings aid in understanding how to balance the advantages of amorphization with potential limitations, such as reduced ductility, to optimize materials for specific mechanical and magnetic applications.

## 2. Materials and Methods

The Cr_16_Mn_16_Fe_16_Co_16_Ni_16_P_20_ alloy was synthesized by arc melting an initial 15 g mixture of high-purity elemental powders: Mn (99.95%), Cr (99.95%), Fe (99.95%), Ni (99.95%), Co (99.95%), and P (98%). The powders were pre-mixed, compacted into a pellet, and arc-melted in pure argon (99.999%). The use of high-purity argon prevented oxidation and other undesirable reactions during melting, ensuring the chemical integrity of reactive elements like phosphorus. Elemental powders (Mn, Cr, Fe, Ni, Co, P) were sourced from Onyxmet Tomasz Olszewski (Onyxmet, Olsztyn, Poland). 

Prior to each melting cycle, including the preliminary one, the furnace chamber was flushed three times by evacuating to 10^−1^ Pa and refilling with high-purity argon at an approximate atmospheric pressure of 10^5^ Pa. This process was followed by melting a 2 g titanium getter to further purify the atmosphere. Each melting cycle was conducted using a non-consumable tungsten electrode (4 mm diameter, ground to a 45–60° angle) powered by an inverter generator with an arc current of 100 A. To stabilize the arc, the electrode was axially ground. Arc melting was conducted using a custom-designed arc furnace manufactured at the Institute of Technical Sciences, University of the National Education Commission, Kraków, Poland, by Krzysztof Ziewiec.

The ingot was re-melted seven times, with each cycle lasting approximately 7 s, for a total of 49 s. After each cycle, the ingot was inverted and mechanically cleaned to ensure homogeneity. Mechanical cleaning involved the removal of any oxide layers or impurities formed during the cooling phase, further improving the compositional accuracy and homogeneity of the alloy. This iterative melting process, combined with mechanical cleaning and the controlled atmosphere, ensured the reproducibility and uniformity of the alloy’s composition and microstructure.

After the final melting, the alloy’s chemical composition was verified by energy-dispersive X-ray spectroscopy (EDS), confirming its consistency with the nominal Cr_16_Mn_16_Fe_16_Co_16_Ni_16_P_20_ composition. EDS measurements were performed at multiple points on the ingot’s surface and cross-section to verify compositional homogeneity and the retention of all alloy elements.

Finally, a 2 g sample was sectioned from the ingot for further testing. Differential scanning calorimetry (DSC) measurements were performed using a Netzsch Jupiter STA449 F3 microcalorimeter (Netzsch, Selb, Germany) under an argon atmosphere (99.999% purity). The heating and cooling rates were set to 20 K/min, and measurements were carried out up to a maximum temperature of 1370 K to capture significant thermal effects and phase transitions. DSC data were used to determine a realistic emissivity coefficient, which enabled the alignment of apparent temperatures from thermography with contact-based measurements, providing more accurate temperature values.

Thermal imaging of the welding process was conducted using a FLIR SC7650 mid-wave infrared camera (FLIR Systems, Wilsonville, OR, USA). The infrared data were captured through a CaF_2_ window with the ResearchIR thermal imaging system and processed on a computer station equipped with both ResearchIR (version 4.40, FLIR Systems, Wilsonville, OR, USA) and Altair software (version 2024, Altair Engineering Inc., Troy, MI, USA). The 2 g ingot was initially melted in an arc furnace, with real-time temperature changes recorded by the infrared camera. Subsequently, a short-duration electric arc was applied directly to the ingot’s surface in air using a TIG-300D PRO 400V UT 26/8 inverter welding machine (Sherman, Warsaw, Poland), with a current pulse of 200 A and a duration of 100 ms. Temperature variations were monitored continuously in a region of interest (ROI) to track phase transformations.

Cooling Conditions: The alloy was subjected to three distinct cooling conditions to study the effects of cooling rates on phase transformations. Slow cooling on a copper plate yielded an average cooling rate of approximately 52 K/s. Accelerated cooling, applied after the short electric pulse, produced an average cooling rate of around 3018 K/s. For rapid cooling, melt-spinning was conducted with a wheel linear speed of 33 m/s, resulting in cooling rates in the range of 10⁵–10⁶ K/s, leading to the formation of an amorphous structure. In the case of melt-spinning, the cooling rate was also influenced by the ribbon thickness, which was approximately 20 µm in this study. The melt-spun ribbons were prepared using a melt spinner (Artvac-Plus, Zabierzów, Poland).

The arc-melted ingot was subsequently cut perpendicularly to the weld line along the cutting plane, and a metallographic specimen was prepared by mechanical polishing and etching in a 3% nitric acid solution in ethanol. Both the cross-sectional surface and the free surface of the ingot were analyzed using a JEOL 6610 SEM equipped (JEOL Ltd., Tokyo, Japan) with an EDS analyzer. Observations were carried out in secondary electron imaging mode at an accelerating voltage of 20 kV and a working distance of 10 mm. Mössbauer spectroscopy measurements were performed in transmission geometry applying the RENON MsAa-4 spectrometer [22] equipped (RENON, Kraków, Poland) with the LND Kr-filled proportional detector and ^57^Co(Rh) source. The absorbers were prepared using about 10 mg/cm² of investigated alloy samples obtained by filing with a diamond file or grinding the ribbon in an agate mortar. Data of the measured spectra were processed using the MOSGRAF software suite (version 2.2, Institute of Nuclear Physics PAN, Kraków, Poland) within the transmission integral approximation. The isomer (center) shifts are reported relative to the α-Fe at room temperature. Errors of reported ^57^Fe Mössbauer parameters are of the order of unity for the last digit shown. 

## 3. Results

**Figure 1:**
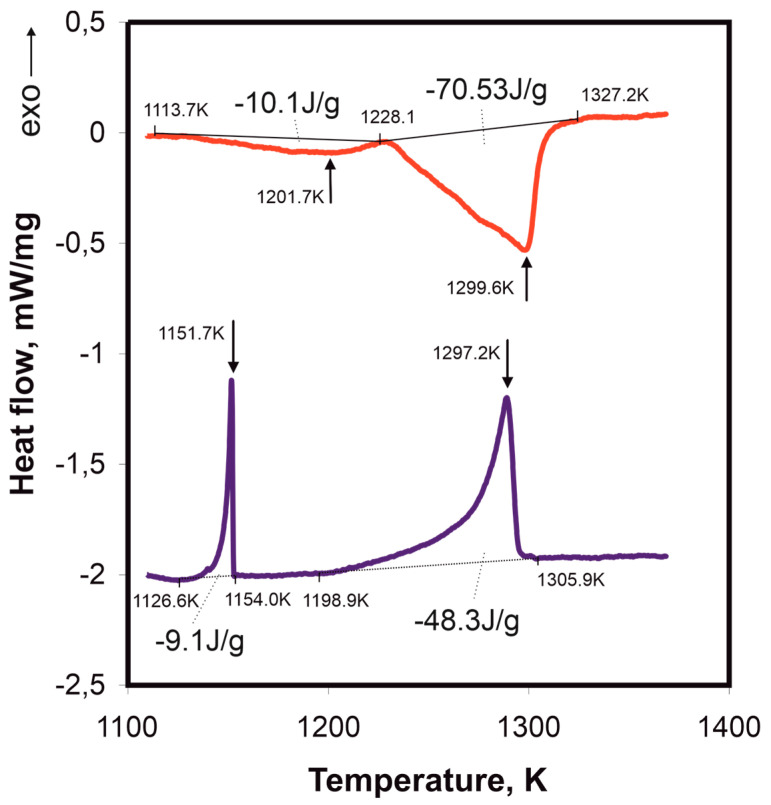
DSC results for the Cr_16_Mn_16_Fe_16_Co_16_Ni_16_P_20_ alloy during heating (red line) and cooling (blue line) at a rate of 20 K/min. The onset and end temperatures of the endothermic and exothermic transitions are marked, along with the corresponding energy values: −10.1 J/g for the range of 1201.7–1228.1 K and −70.53 J/g for 1299.6–1327.2 K during heating, and −9.1 J/g and −48.3 J/g for cooling at 1151.7 K and 1297.2 K, respectively.

Figure 1 presents the DSC analysis results for the Mn_16_Cr_16_Ni_16_Fe_16_Co_16_Ni_16_P_20_ alloy during heating and cooling. During heating (red curve), two distinct endothermic peaks are observed. The first peak starts at 1113.7 K, reaches a maximum at 1201.7 K, and ends at 1228.1 K, with an absorbed energy of −10.1 J/g. The second, more intense endothermic peak begins at 1297.7 K and ends at 1327.2 K, corresponding to the melting process, with an energy absorption of −70.53 J/g. During cooling (blue curve), two exothermic peaks appear, indicating crystallization processes. The first peak starts at 1126.6 K, reaches a maximum at 1154.0 K, and ends at 1198.9 K, with a released energy of −9.1 J/g. The second, more pronounced exothermic peak begins at 1198.9 K, reaches a maximum at 1297.2 K, and ends at 1305.9 K, releasing −48.3 J/g, suggesting the crystallization of the primary phase from the liquid.

**Figure 2:**
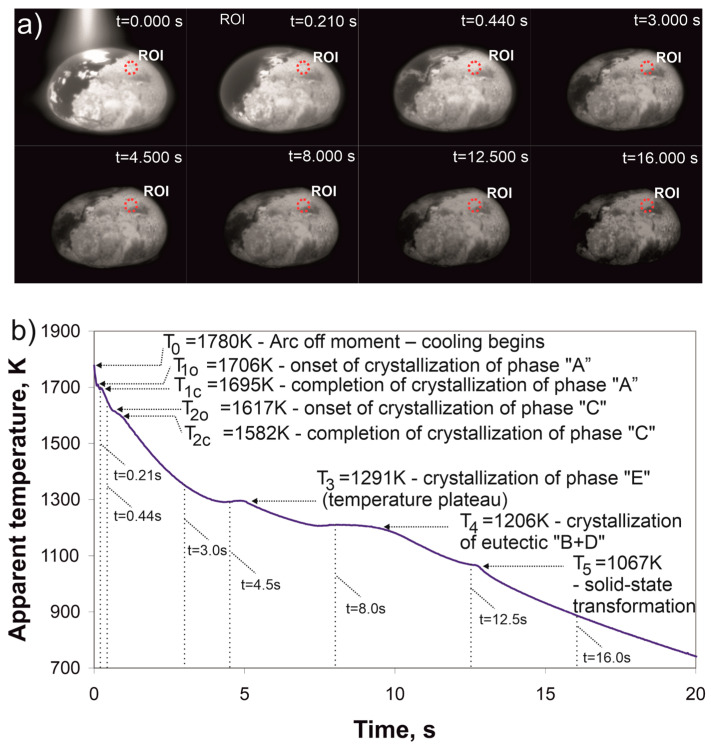
(**a**) Thermal images of the arc-melted ingot, with the region of interest (ROI) marked in purple. The image at t = 0.000 s shows the ingot immediately after the extinction of the electric arc. Subsequent images depict the cooling process over time. The temperature-time curve for the ROI is shown in (**b**), indicating key phase transitions: solidification of phase A at T_1_ = 1695 K, precipitation of phase C at T_2_ = 1623 K, phase E crystallization at T_3_ = 1291 K, eutectic crystallization at T_4_ = 1206 K, and a solid-state transformation at T_5_ = 1067 K. Cooling after arc melting on a copper plate (52 K/s).

Figure 2 presents the results of the thermographic measurements of the Cr_16_Mn_16_Fe_16_Co_16_Ni_16_P_20_ alloy ingot during cooling after the electric arc was turned off, with the ingot placed on a copper plate. At t_0_ = 0.00 s, when the arc was extinguished, the cooling begins from an initial temperature of T_0_ = 1780 K. Following this phase, there is a brief slowdown in cooling, starting at a temperature of T_1o_ = 1706 K and ending at T1c = 1695 K. Below this temperature, the next segment of the curve is characterized by an increased cooling rate (t = 0.44 s), followed by another slowdown, associated with the precipitation of a new phase, which constitutes the second stage of crystallization. This stage begins at T_2o_ = 1617 K and ends at T_2c_ = 1582 K. Below this temperature, the sample cools continuously, initially at a higher rate, which then slows down (t = 3.0 s), followed by a clear temperature plateau at T_3_ = 1291 K. The temperature of the ingot then decreases to T_4_ = 1206 K, where another temperature plateau lasting almost 1.5 s is observed. After approximately 10 s from the start of cooling and up to t = 12.5 s, the temperature continues to decrease steadily. At T_5_ = 1067 K, another peak occurs, related to a subsequent phase transformation.

**Figure 3:**
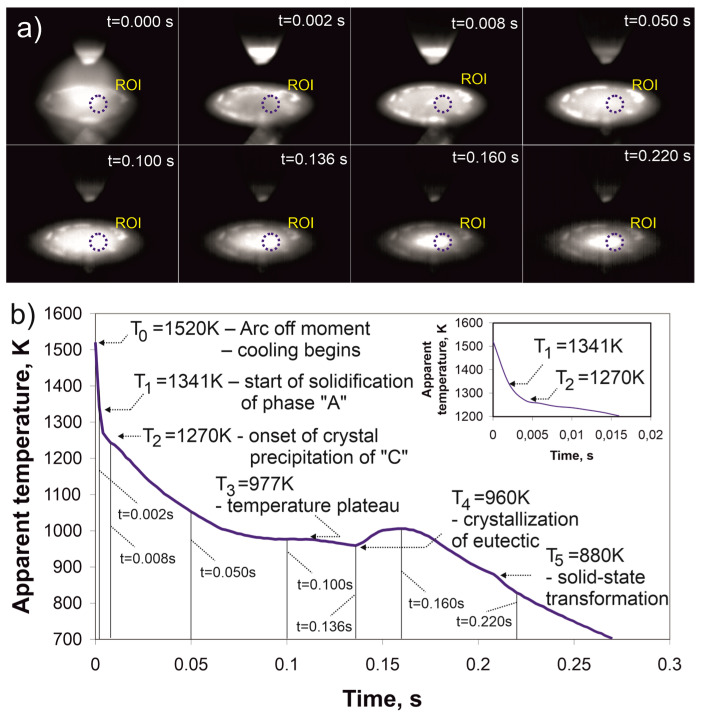
(**a**) Thermal images of the ingot with the region of interest (ROI) marked in blue, captured during rapid cooling after a short electric pulse. The image at t = 0.000 s shows the moment the arc was extinguished. The temperature-time curve in (**b**) highlights key thermal events: solidification at T_1_ = 1341 K, precipitation at T_2_ = 1270 K (magnified in the inset), a plateau at T_3_ = 977 K, a local minimum at T_4_ = 960 K, and a solid-state transformation at T_5_ = 880 K, visible as a small peak. Cooling after short electric pulse (3018 K/s).

Figure 3 presents the thermographic measurement results of the Cr_16_Mn_16_Fe_16_Co_16_Ni_16_P_20_ alloy ingot during rapid cooling after heating by a single current pulse lasting 100 ms. At t_0_ = 0.00 s, when the current pulse was turned off, the initial temperature was T_0_ = 1520 K, marking the start of cooling. After this phase of rapid temperature decrease, the cooling rate temporarily slows down, which may be associated with the onset of phase precipitation at T_1_ = 1341 K. Between T_1_ = 1341 K and T_2_ = 1270 K, the cooling rate decreases further, indicating the initiation of another transformation. Upon reaching the temperature of T_3_ = 977 K, a temperature plateau occurs. Subsequently, the cooling curve reaches a local minimum at T_4_ = 960 K. Finally, the temperature drops below T_5_ = 880 K, where another phase transformation occurs.

**Figure 4:**
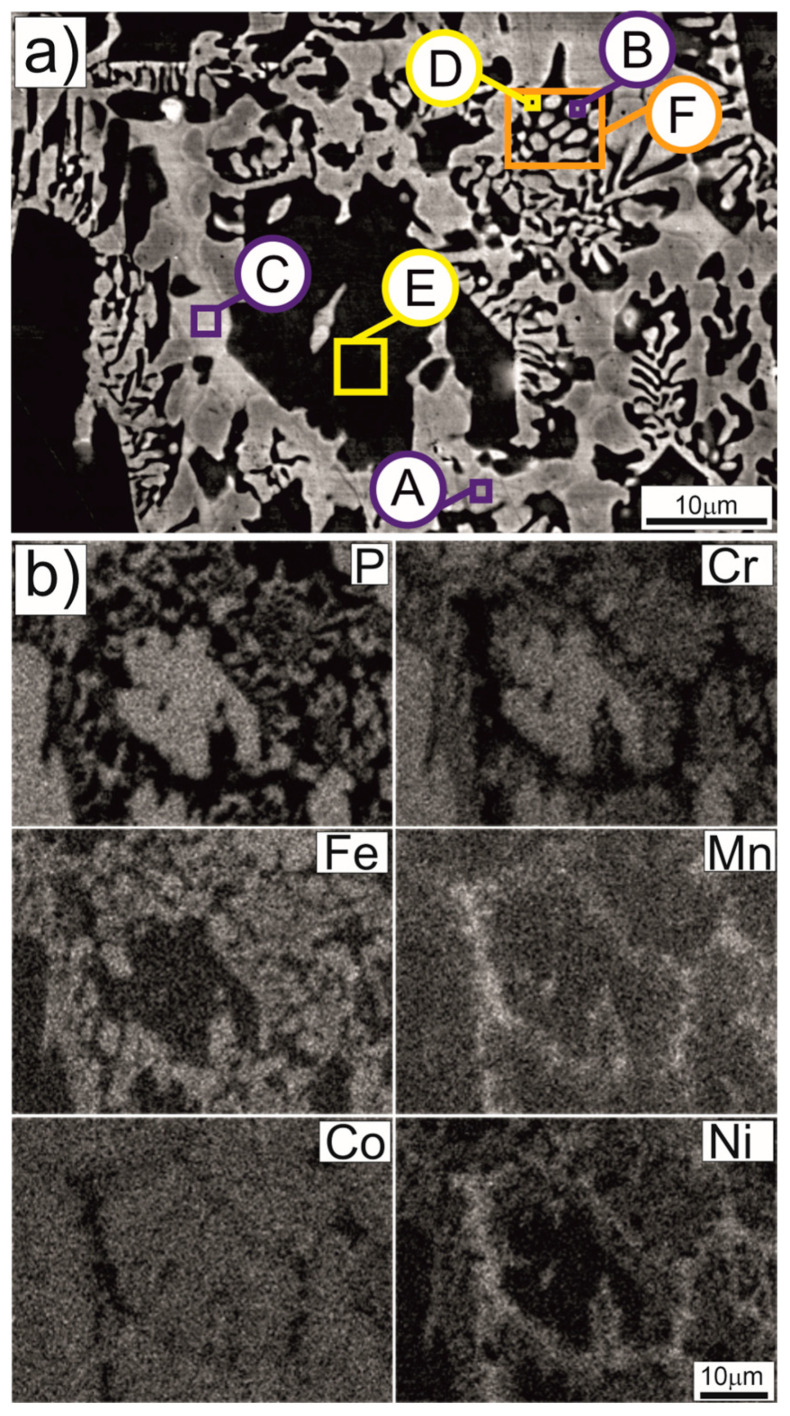
Microstructure of the Cr_16_Mn_16_Fe_16_Co_16_Ni_16_P_20_ alloy ingot after arc remelting. SEM image (**a**) and elemental distribution maps (**b**) obtained from EDS analysis for elements P, Cr, Fe, Mn, Co, and Ni. The letters on image (**a**) correspond to phase and microstructural components described in Table 1. Sample preparation: Metallographic specimens were mechanically polished and etched using a 3% nitric acid solution in ethanol. SEM observations were performed at an accelerating voltage of 20 kV.

**Figure 5:**
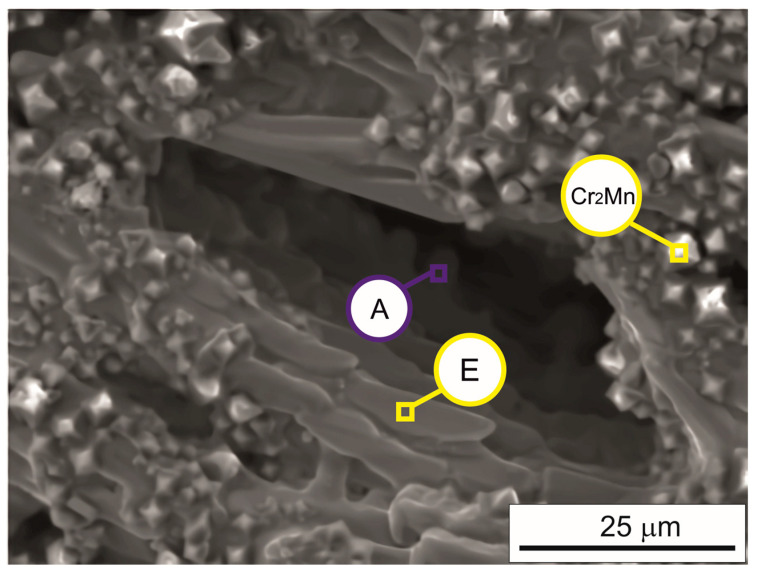
SEM image of the free surface of the ingot after arc remelting and cooling in a pure argon atmosphere. Dendrites of phase “A”, elongated crystals of phase “E”, and small precipitates of a Cr_2_Mn-like phase with a square-based pyramidal morphology are visible. Sample preparation: Metallographic specimens were mechanically polished and etched using a 3% nitric acid solution in ethanol. SEM observations were performed at an accelerating voltage of 20 kV.

Figure 4a shows the microstructure of the Cr_16_Mn_16_Fe_16_Co_16_Ni_16_P_20_ alloy ingot after arc remelting, obtained using SEM. The SEM image identifies phase and structural components with diverse morphologies, as listed in Table 1. Component A exhibits a weakly developed dendritic morphology, while Component B is the brighter component of the rod-like eutectic. Component C fills the spaces between the eutectic regions and faceted crystals. Component D represents the darker phase of the rod-like eutectic, and Component E has a morphology resembling faceted crystals with the highest phosphorus content. Figure 5 highlights additional features on the free surface of the ingot, such as the pyramid-like morphology of the intermetallic compound Cr_2_Mn.

**Figure 6:**
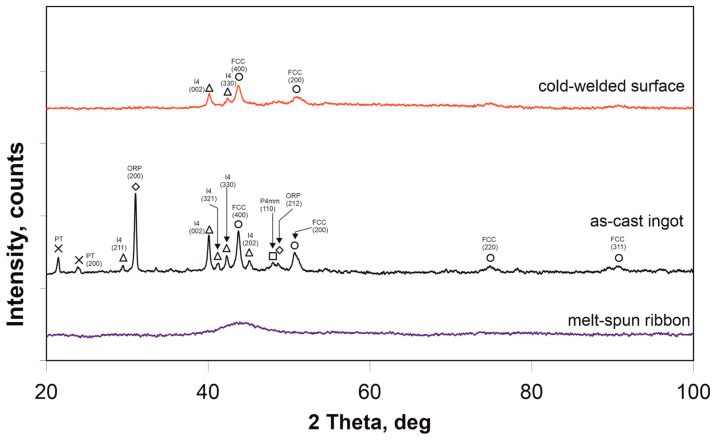
XRD patterns of the Cr_16_Mn_16_Fe_16_Co_16_Ni_16_P_20_ alloy in three different processing states: arc-melted ingot (**middle**), cold-welded surface (**top**), and melt-spun ribbon (**bottom**). The diffraction peaks correspond to FCC, tetragonal (I4), and orthorhombic (ORP) phases. The melt-spun ribbon shows a nearly amorphous structure. XRD test conditions: Scans were performed in the 2θ range of 20°–90° with a step size of 0.02°, at a voltage of 40 kV and a current of 30 mA.

Figure 6 shows XRD patterns of the Mn_16_Cr_16_Ni_16_Fe_16_Co_16_P_20_ alloy in three different processing states: arc-melted ingot, surface heated by a short electric pulse, and melt-spun ribbon. For the arc-melted ingot, several phases were identified, including FCC, tetragonal, and orthorhombic phases. The sample heated by a short electric pulse primarily exhibited FCC and tetragonal phases, while the melt-spun ribbon showed a broad intensity maximum, indicating an amorphous structure.

**Figure 7:**
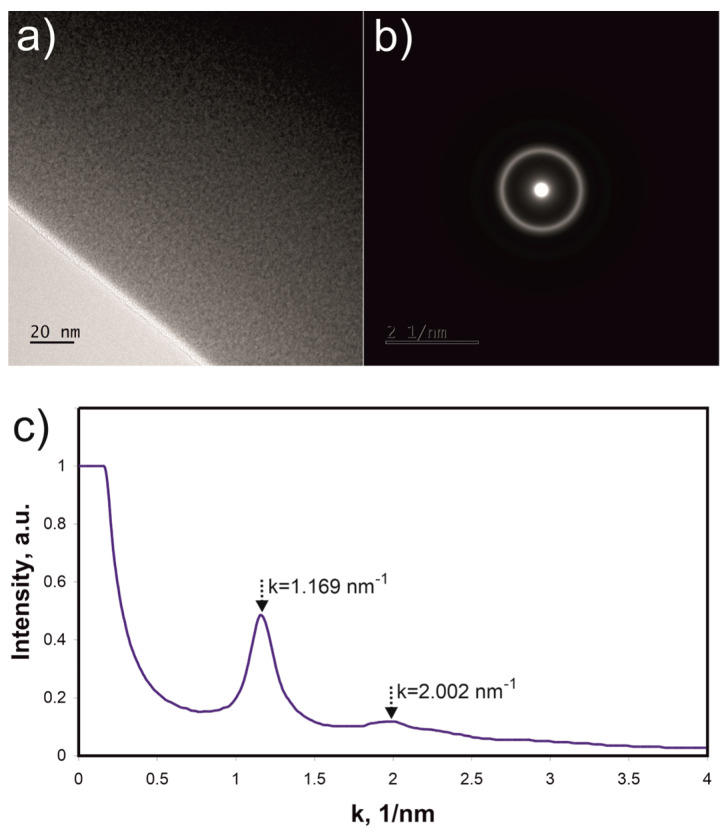
(**a**) TEM image showing the amorphous microstructure of the Cr_16_Mn_16_Fe_16_Co_16_Ni_16_P_20_ alloy ribbon after melt-spinning. (**b**) Diffraction pattern with rings, indicating a disordered atomic structure. (**c**) Intensity profile of the diffraction rings, with two peaks identified at wave vector values of k = 1.169 nm^−1^ and k = 2.002 nm^−1^, confirming the amorphous nature of the sample.

Figure 7 presents the TEM results for the melt-spun ribbon. The TEM micrograph reveals a homogeneous, grain-free microstructure characteristic of amorphous materials. The selected area electron diffraction (SAED) pattern confirms the disordered atomic arrangement, while the intensity profile of the diffraction rings reflects local short-range ordering typical of transition metal-phosphorus alloys.

**Figure 8:**
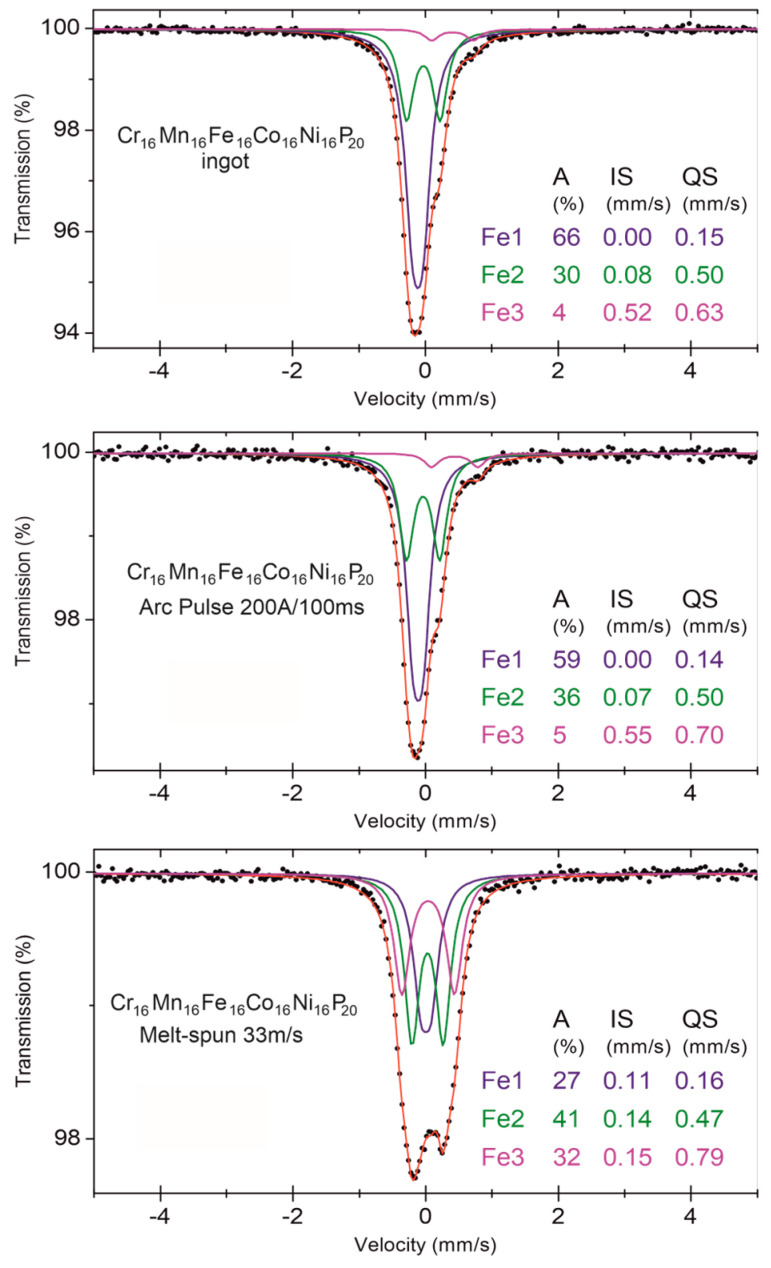
Mössbauer spectra for Cr_16_Mn_16_Fe_16_Co_16_Ni_16_P_20_ in different processing states: as-cast ingot (**top**), after short arc pulse (**middle**), and melt-spun ribbon (**bottom**). The spectra were fitted with three components corresponding to different local environments around iron atoms, labeled as Fe1, Fe2, and Fe3. The table includes the relative area (A), isomer shift (IS), and quadrupole splitting (QS) for each component.

Mössbauer spectroscopy provides detailed insights into the local atomic environment and electronic states of iron atoms in the studied material (Figure 8). The spectra for all processing states, including the as-cast ingot, the surface modified by a short electric pulse, and the melt-spun ribbon, confirmed the paramagnetic state of iron. Isomer shift (IS) values close to 0 mm/s in the as-cast ingot suggest a metallic, paramagnetic environment for iron atoms. The quadrupole splitting (QS) of 0.15 mm/s indicates a symmetric local electric field, characteristic of the FCC phase, which is the dominant phase in this state.

For the sample subjected to a short electric arc pulse, slight changes in the Mössbauer spectrum were observed, with a reduced full width at half maximum (FWHM) of 0.27 mm/s, indicating minor structural ordering. The relative contributions of spectral components remained largely unchanged, suggesting that the local chemical and crystallographic environment around the iron atoms remained consistent with the as-cast state.

In contrast, the Mössbauer spectrum of the melt-spun ribbon showed significant changes. The higher QS values, up to 0.79 mm/s, reflect increased asymmetry in the electric field, indicative of lower structural symmetry or higher chemical disorder. This result aligns with the amorphous structure observed in the TEM and XRD analyses. The broadened spectral lines and higher FWHM values indicate greater structural inhomogeneity, consistent with the rapid cooling process during melt-spinning.

## 4. Discussion

This study provides a detailed investigation into the phase transformations and microstructural evolution of the Cr_16_Mn_16_Fe_16_Co_16_Ni_16_P_20_ alloy under various cooling conditions. The DSC analysis highlights the distinct behavior of first-order phase transitions, characterized by hysteresis where crystallization temperatures are consistently lower than melting temperatures. The weaker thermal anomaly during crystallization, compared to melting, confirms the typical behavior of such transitions [23]. The heat associated with the dissolution and crystallization of phase “E” is notably higher than that of eutectic transformations, aligning with findings on multicomponent eutectic systems where melting temperatures decrease with increasing alloy complexity [24].

Thermographic analysis revealed significant differences in the cooling dynamics between slow cooling on a copper plate and rapid cooling induced by short arc pulses. The observed transformation temperatures, such as FCC phase crystallization at 1706 K during slow cooling and 1341 K during rapid cooling, underscore the role of cooling rates in shifting transformation temperature. Additionally, the plateau temperatures T_3_ (1291 K and 977 K) and T_4_ (1206 K and 960 K) highlight the transitions linked to the crystallization of faceted crystals and the formation of eutectic constituents, respectively.

High-resolution TEM imaging and XRD analysis confirmed the amorphous nature of the ribbon produced via melt-spinning. The intensity profile of the diffraction rings observed in TEM, with two broad peaks at wave vector values of k = 1.169 nm^−1^ and k = 2.002 nm^−1^, corresponds to characteristic features of reciprocal space scattering in amorphous structures. These values are indicative of the short-range atomic ordering typical of metallic glasses based on transition metals and phosphorus. The diffuse peaks in this study are consistent with XRD results, which revealed a broad intensity maximum characteristic of amorphous structures. Similar diffuse diffraction patterns and broad peaks in reciprocal space have been reported in [25] for other amorphous systems, further validating the successful amorphization achieved through rapid cooling during the melt-spinning process.

Phosphorus significantly influences the amorphization tendency of the studied Cr_16_Mn_16_Fe_16_Co_16_Ni_16_P_20_ alloy. Despite the presence of elements such as Mn and Cr, which typically favor the formation of intermetallic compounds due to their strongly negative mixing enthalpies with phosphorus (−49 kJ/mol for Mn-P and −41 kJ/mol for Cr-P), the alloy exhibits the ability to amorphize directly from the liquid state. This behavior underscores the unique role of phosphorus in disrupting crystallization and enhancing glass-forming ability [26].

The role of phosphorus in promoting phase stability and modifying solidification pathways is further supported by phase diagram analysis. Binary [27] and ternary [28] phase diagrams indicate that phosphorus addition promotes the formation of phosphide phases, which appear as independent intermediate phases or within eutectic systems. Moreover, phosphorus significantly reduces the solidification temperature range of primary, phosphorus-poor phases. Cooling profiles recorded during solidification, both after remelting on a copper plate and following a brief electric arc pulse, suggest the presence of low-phosphorus phases, such as phase “A”, and phosphide-rich phases, including those forming in eutectic (“B” + “D”) or non-eutectic systems (“C” and “E”). The obtained results indicate that phosphorus plays a significant role in shaping the crystallization process and the resulting microstructure; however, its influence should be considered in the context of interactions with other elements present in the complex multicomponent system.

The cooling profile recorded during welding (at a cooling rate of 154 K/s) demonstrates that the Cantor alloy solidifies at a temperature of 1478 K [29]. Previous studies reported its melting temperatures as 1607 K [30,31], 1523 K [32], and 1543 K [33], indicating a relatively narrow range of solidification. In contrast, the Cr_16_Mn_16_Fe_16_Co_16_Ni_16_P_20_ alloy containing phosphorus exhibits a significantly broader solidification temperature range and more complex crystallization behavior. Pronounced thermal effects were observed at T_3_ = 1291 K and T_4_ = 1206 K during slow cooling on a copper plate (52 K/s), and at T_3_ = 977 K and T_4_ = 960 K during rapid cooling following a brief electric arc pulse (3018 K/s). This marked reduction in solidification temperatures compared to the Cantor alloy demonstrates the role of phosphorus in modifying thermal behavior and promoting amorphization.

These results highlight the critical importance of controlling cooling rates and alloy composition to optimize phase stability and microstructure. The ability to achieve amorphous structures through rapid cooling opens up opportunities for applications requiring high wear resistance, thermal stability, and optimized magnetic properties.

## 5. Conclusions

1. Cooling rates significantly influence the microstructure and phase composition of the Cr_16_Mn_16_Fe_16_Co_16_Ni_16_P_20_ alloy. Slow cooling after arc melting results in a complex microstructure comprising multiple crystalline phases such as FCC “A”, tetragonal “B”, “C”, “D”, and orthorhombic “E”, while faster cooling after a short electric arc pulse reduces the number of phases to primarily FCC “A” and tetragonal “D”. Very rapid cooling using the melt-spinning method results in an amorphous structure, as confirmed by XRD and TEM analyses.
2. Phosphorus addition enhances the alloy’s glass-forming ability during rapid cooling. The amorphous structure observed in the melt-spun ribbon demonstrates that the combination of phosphorus and high mixing entropy promotes the formation of amorphous phases during extremely fast cooling. However, further studies are necessary to understand the influence of phosphorus on mechanical properties and limitations, such as reduced ductility.
3. Thermal analysis revealed specific phase transformation temperatures for different phases: FCC “A” crystallizes between 1706 K and 1695 K, phase “C” between 1617 K and 1582 K, orthorhombic phase “E” at approximately 1291 K, and the eutectic crystallization of phases “B” and “D” occurs at around 1206 K. Higher cooling rates significantly increase undercooling, shifting transformation temperatures downward by up to 365 K.
4. Phase transformations following short electric arc pulses exhibited distinct characteristics compared to slow cooling on a copper plate. Specifically, undercooling effects were more pronounced, and transformations such as FCC phase crystallization occurred at significantly lower temperatures (e.g., 1341 K compared to 1706 K). These results highlight the dynamic nature of phase formation under non-equilibrium cooling conditions induced by short arc pulses.
5. Mössbauer spectroscopy demonstrated the influence of cooling rates on the local atomic environment of iron. In slowly cooled samples, iron predominantly exists in a paramagnetic state within the FCC phase, while faster cooling increases structural heterogeneity and electric field asymmetry. In the amorphous ribbon, parameters such as quadrupole splitting (up to 0.79 mm/s) indicate significant chemical disorder, confirming amorphization.
6. Phosphorus-rich phases, such as tetragonal “C” and orthorhombic “E”, crystallize at lower temperatures compared to the phosphorus-poor FCC “A”. The phase transformation sequence reflects the key role of phosphorus in lowering crystallization temperatures and promoting eutectic formation.
7. The observed phase transformations highlight the potential of phosphorus-enriched high-entropy alloys for applications requiring wear resistance and thermal stability. Precise control of cooling rates and alloy composition is essential to balance the benefits of amorphization while maintaining good ductility for advanced applications.

## Figures and Tables

**Table 1:** Phase components identified by XRD and corresponding microstructural components identified based on SEM/EDS from the arc-melted ingot.

No.	XRD	Morphology	Chemical Composition Based on EDS, % at.					
			Cr	Mn	Fe	Co	Ni	P
1	FCC, Fm-3m (225)	Gray grains (dendrites) “A”	6.94	19.40	26.36	17.32	28.34	1.64
2	Tetragonal, P	Eutectic; brigh component “B”	10.76	15.28	26.91	18.80	21.87	6.38
3	Tetragonal, P4/mmm (123)	Light areas “C”	1.55	32.51	5.97	7.85	39.78	12.33
4	Tetragonal, I-4 (82)	Eutectic; dark component “D”	20.09	11.76	12.27	17.15	9.85	28.87
5	Orthorhombic, Pnma (62)	Dark faceted crystals “E”	24.89	8.31	9.61	17.33	6.90	32.97
6	Eutectic (P & I-4)	Rod-like eutectics “F”	15.25	13.30	21.21	18.50	15.74	16.00

The chemical composition values reflect the precision of the EDS analysis, with two decimal places indicating a resolution better than ±0.4%, consistent with the calibration and averaging methods used.

## Data Availability

The raw data supporting the conclusions of this article will be made available by the authors on request.
